# Changes of Corneal Biomechanical Properties upon Exclusive Ytt-/Sr-90 Irradiation of Pterygium

**DOI:** 10.3390/s21030975

**Published:** 2021-02-02

**Authors:** Fritz Rigendinger, Daniel M. Aebersold, Zeljka Cvejic, Bojan Pajic

**Affiliations:** 1 Eye Clinic Orasis, Swiss Eye Research Foundation, 5734 Reinach AG, Switzerland; rigendinger@gmx.ch; 2 Department of Radiation Oncology, Inselspital, Bern University Hospital, University of Bern, 3012 Bern, Switzerland; Daniel.Aebersold@insel.ch; 3 Department of Physics, Faculty of Sciences, University of Novi Sad, Trg Dositeja Obradovica 4, 21000 Novi Sad, Serbia; zeljka.cvejic@df.uns.ac.rs; 4 Division of Ophthalmology, Department of Clinical Neurosciences, Geneva University Hospitals, 1205 Geneva, Switzerland; 5 Faculty of Medicine of the Military Medical Academy, University of Defense, 11000 Belgrade, Serbia

**Keywords:** pterygium, beta irradiation, corneal aberrations

## Abstract

Background: It is known that pterygia above a certain size cause astigmatism and other aberrations of the human cornea and thus impair the quality of vision. Exclusive Sr-/Ytt-90 beta irradiation is a highly effective treatment for primary pterygia. The aim of this retrospective study is to determine the extent to which higher order corneal aberrations are affected by this treatment. Methods: Evaluation of corneal topographies and wavefront aberration data of 20 primary pterygia patients generated before and at different points in time in the first year after irradiation. Additionally, the size of the pterygium was measured. Results: The study showed a significant increase in coma and triple leaf aberrations in pterygia with a horizontal length of 2 mm and more. It was also found that a pterygium size greater than 2 mm significantly induces astigmatism. Both phenomena reduce visual quality. In none of the patients could a pterygium recurrence be detected after irradiation. Conclusions: If the pterygium size is less than 2 mm, early exclusive Sr/Ytt-90 beta irradiation can be recommended. If the size is more than 2 mm, a pterygium excision 6 months after beta irradiation can be discussed.

## 1. Introduction

**Figure 1:**
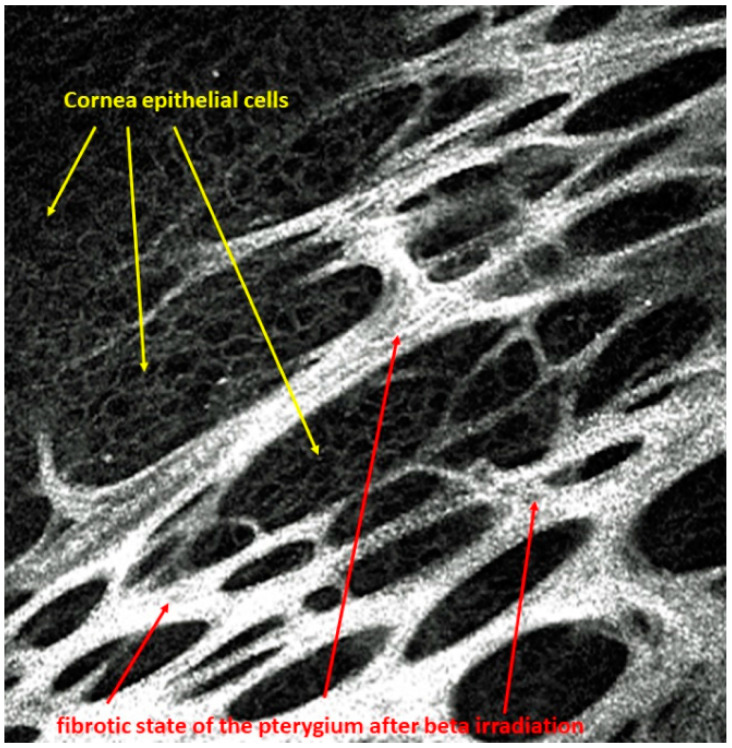
Islets of corneal epithelial cells arranged within the fibrous structures of the pterygium after beta irradiation.

A pterygium grows proliferatively toward the center of the cornea with Bowman’s membrane as its guiding structure, which is destroyed [1,2]. A narrow zone at the head of the pterygium is vascular-free, while the base is vascular-rich. The base covers the peripheral cornea. However, the pterygium can be undermined. In particular, a confocal laser microscope can detect complete epithelial islands under the fibrous pterygium structures, as shown in Figure 1.

**Figure 2:**
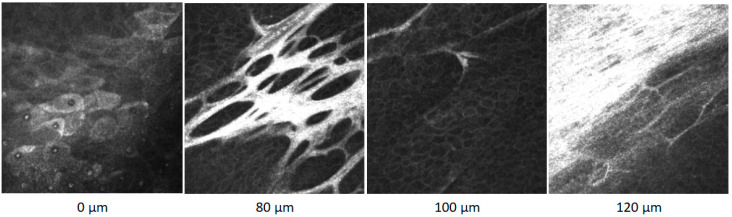
Confocal microscope images of a pterygium within the surface at 0 µm, through layers in deeper positions at 80 and 100 µm to the sclera at 120 µm.

After beta irradiation, the pterygium appears as an avascular, fibrotic membrane, which is very sharply demarcated from the stromal structure of the cornea. The pterygium is fixed like a honeycomb to the pterygium head and to the stromal part in the area of the pterygium bed. Partially, the pterygium can be undermined and is lined with epithelial cells. By means of confocal microscopy, the layers of a pterygium can be visualized very well, from the superficial epithelial cells to the fibrotic structure of the pterygium with epithelial islands at a depth of 80 µm. At a depth of 100 µm, the underminable part is lined with epithelial cells reaching down to the sclera, which begins here at 120 µm (Figure 2). In a case series, it could be shown that on average, the pterygium thickness decreases by 40 μm after beta irradiation compared to the active, non-treated state. An average prominence of 120 μm remains after beta irradiation [3].

Clinically, the pterygium initially causes irritation and dry eye disease by interrupting the tear film. In later stages, the growing tissue causes astigmatism by flattening the cornea in the direction of the growing pterygium. This occurs mostly horizontally, until finally, the visual axis is directly affected, all of which can lead to reduced visual acuity.

Recent meta studies estimated global prevalence of pterygium at 10–12% [4,5]. Exposure to ultraviolet light is thought to be the primary risk factor and corresponds well with the fact that pterygium is more often found in tropical regions [6,7,8]. Other reported risk factors include increasing age, male sex, living in rural areas, oncogenic virus infection, loss of heterozygosity and short axial length [5,9,10,11,12]. The etiology of the pterygium is not fully understood. An interaction of different pathological factors is likely [13,14]. The initial damage is a focal alteration of the limbal stem cells due to chronic exposure to ultraviolet light. The growth factors FGF (fibroblast growth factor), VEGF (vascular endothelial growth factor), TGF (transforming growth factor) and SCM (stem cell factor) are found in pterygia [15,16]. FGF and TGF are, among other things, responsible for the proliferation of fibroblasts and for increased production of the extracellular matrix. VEGF has an angiogenetic effect, while SCM is responsible for the regulation of mast cells. One method to treat pterygia is by directly inhibiting these growth factors. The low level of IGFBP3 (insulin-like growth factor binding protein 3) in pterygium suggests that growth proliferation is not controlled analogous to tumor cells [17].

**Figure 3:**
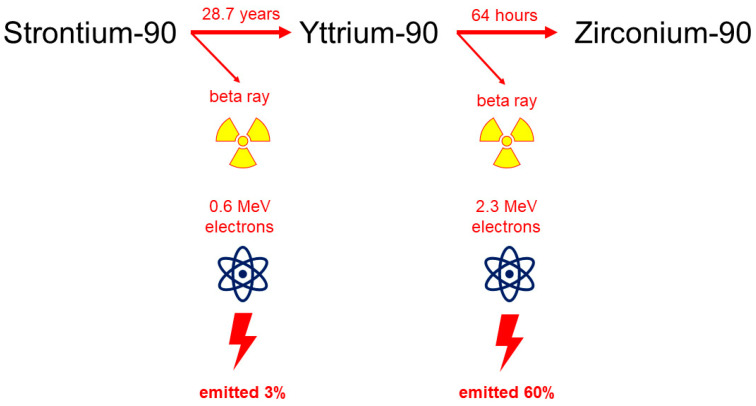
Stronium-/Yttrium-90 beta-irradiation decay scheme.

Exclusive resection of the pterygium known as the bare sclera technique is associated with high recurrence rates; therefore, different adjuvant therapies including conjunctival autografting combined with the application of anti-proliferative/anti-inflammatory substances (mitomycine-C, 5-fluorouracil, anti-VEGF, ciclosporin A) or irradiation have been tried or are still used today [18,19]. Adjuvant beta-irradiation brachytherapy in the postoperative treatment of pterygium dates back to the late 1940s and has been covered extensively in the literature [20,21]. It is also well known that beta radiation consists of high-speed electrons, which are rapidly attenuated by biological tissues (2 MeV beta particles have a range of a few mm in water with a very steep dose gradient below surface). This makes it very useful for surface radiation treatments where deep penetration into tissue is undesirable. The less known exclusive Strontium-/Yttrium-90 beta irradiation without surgery with a total dose of 36 Gy in six fractions has shown to be an efficient, and to date, recurrence-free and safe treatment against primary or recurrent pterygia that leaves an inactive, avascular pannus reduced in size [22,23,24]. Strontium ^90^Sr has a decay half-life of 28.7 years and decays to yttrium ^90^Y with the emission of a spectrum of beta particle energies to a maximum of 0.6 MeV. Then, ^90^Y itself decays to stable zirconium-90 with a half-life of 64 h and yielding beta particles to a maximum energy of 2.3 MeV which are used for therapeutic treatments, as shown in Figure 3.

As a result, the initial higher dose could be reduced to a current total dose of 36 Gray in six fractions [22,23,24].

As mentioned before, pterygia induce astigmatism and other ocular aberrations to a degree that correlates with pterygium size [25,26,27]. Pajic et al. noted a further increase in astigmatism after exclusive irradiation and attributed this to pulling forces by the retracting pterygium [28]. Because of this and the potential induction of other corneal aberrations, a secondary excision can become necessary to improve visual quality.

By careful analysis of corneal topographies, this retrospective study intends to give a better understanding of the changes of corneal aberrations after exclusive Strontium-/Yttrium-90 beta irradiation in order to optimize current treatment.

## 2. Materials and Methods

Between 2001 and 2019, 20 eyes from 19 patients (8 male and 11 female, mean age 57 ± 9.9 years, 38 to 74 years old, 8 right and 12 left eyes) with primary pterygia were treated by exclusive Strontium-/Yttrium-90 beta irradiation in the form of brachytherapy. Exclusion criteria were recurrent pterygia, pseudopterygia, coexisting other corneal or scleral pathologies including dry eye disease, operation and trauma and contact lens wearing. The protocol (2020-00907) of the study was approved by the Ethics Committee Nordwest- und Zentralschweiz (EKNZ, Switzerland).

**Figure 4:**
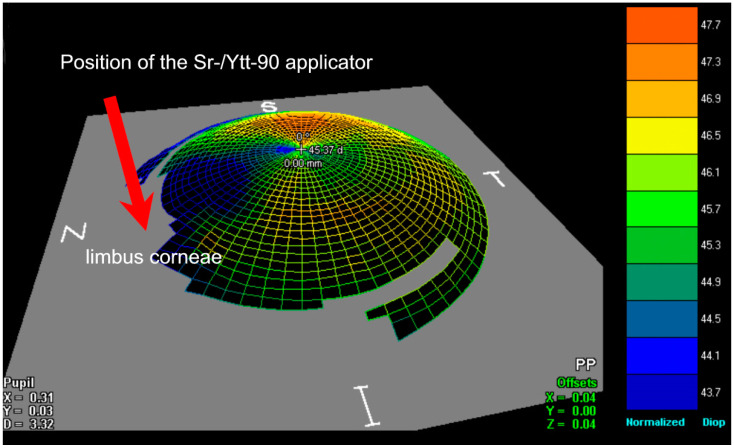
Topographical, schematic illustration shows the location on the nasal corneal limbus where the radiation applicator was placed. Note the cornea flattens in the area of the pterygium.

The cumulative irradiation dose was 36 Gray for each pterygium, equally split into six fractions of six Gray administered twice weekly for three consecutive weeks. The irradiation source was a Strontium-90/Yttrium-90 probe, exclusive β⁻ particle emitting radionuclides, contained in a 12 mm diameter plate. The application opening was covered with a layer of 0.002 mm thickness steel and a layer of 0.01 mm thickness aluminum. This filter reduced the strontium-90 radiation to 3% and the yttrium-90 radiation to 60% of the initial value. The bremsstrahlung dose rate contribution of Sr-90 itself was far lower than the bremsstrahlung dose rate from Y-90, due to differences in their beta-endpoint energies. In addition, bremsstrahlung dose rates induced by beta sources within lead or alumina are negligible [29]. An applicator was applied directly to the pterygium using a pen-like holder, with the center of the radiation source directed towards the corneal limbus, as shown in Figure 4.

**Figure 5:**
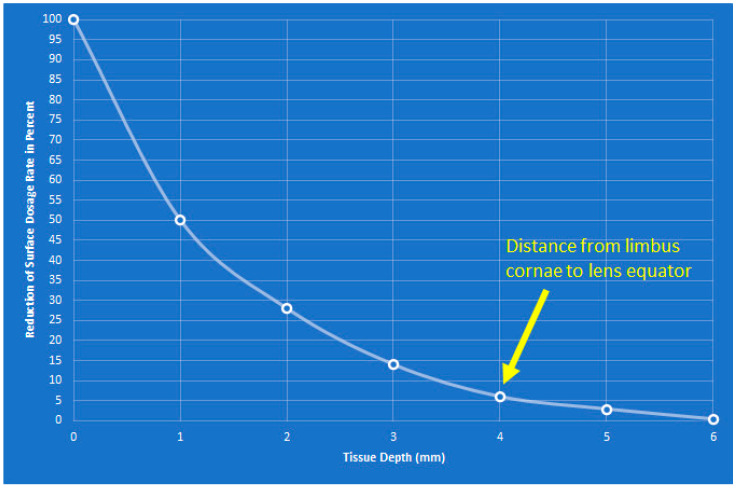
The diagram shows the penetration depth of beta radiation in the tissue. It can be seen that only 6% of the irradiation penetrates 4 mm, i.e., to the equator of the lens.

Beta irradiation has the advantage that most of its energy is absorbed in superficial tissue with only around 6% reaching 4 mm depth; thus, cataract formation as a side effect is highly unlikely [30]. The percentage reduction of the surface dosage rate of the Sr-/Ytt-90 applicator was obtained by testing the polystyrene absorber as a tissue equivalent [31] (Figure 5).

While mild and self-resolving symptoms like redness and irritation were frequent, no severe side effects were observed. After application of beta irradiation, the vascularization and thus also the activity of the pterygium decreased continuously and significantly until an avascular, flat, grey membrane remained. This was achieved after 3 months. This dynamic was observed in all patients. The procedure was performed at the Department of Radio Oncology at Bern University Hospital, Switzerland.

To assess changes in corneal aberrations, corneal topographies were performed as part of the clinical follow-up before and after irradiation. Appointments occurred on a regular basis, but the intervals varied between the individual patients. Clinical follow-up consisted of a full ophthalmic examination including assessment of visual acuity, subjective refraction and measurement of horizontal pterygium size.

In 9 eyes, corneal topographies were obtained using the Orbscan II (Bausch & Lomb, USA), which combines vertical slit-scanning with a Placido disk and acquires elevation and curvature data directly from both the anterior and posterior corneal surface. Of interest were not only the astigmatism power and its axis but also the irregularities in the central 3.0- and 5.0-mm cornea zones. Values from before and after irradiation were compared; for 5 of the pterygia, a comparison between several time points was possible.

**Figure 6:**
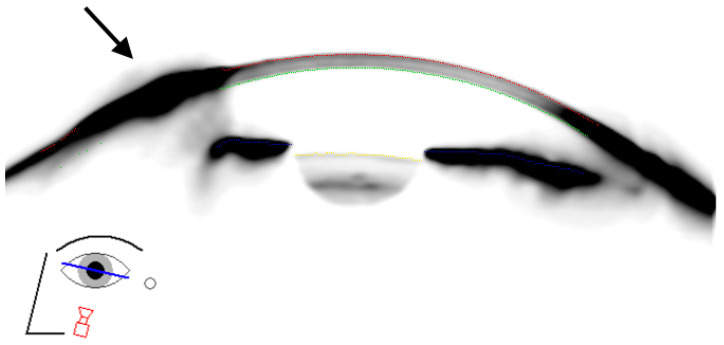
A Pentacam–Scheimpflug image (inverted colours) shows a nasal pterygium in form of corneal thickening (arrow) of the left eye of a 62-year-old male with a horizontal length of 3.5 mm before irradiation. By merging 25 Scheimpflug images, the 3D model of the anterior eye segment and all other calculations are generated.

**Figure 7:**
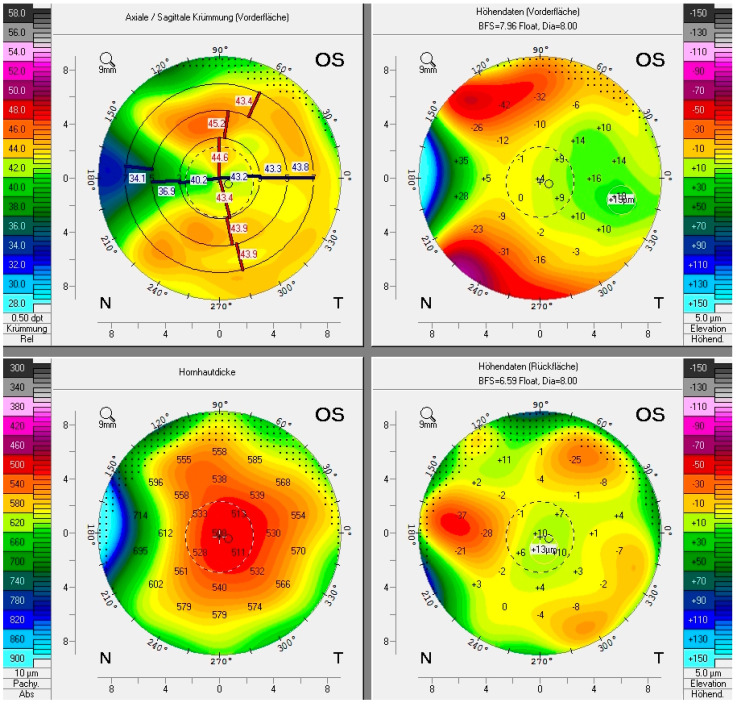
A typical Pentacam display of the same nasal pterygium shown in Figure 5. Clockwise starting from top left: anterior sagittal curvature map, anterior elevation map, posterior elevation map, corneal thickness map (pachymetry). The pterygium thickens and flattens the peripheral nasal cornea and thus reduces local refractive power.

In the other 11 eyes, corneal tomographies were acquired using the Pentacam (Oculus Optikgeräte, Wetzlar, Germany), a non-invasive rotating Scheimpflug camera, which calculates a 3D virtual model of the anterior eye segment by processing 25,000 distinct elevation points on the anterior and posterior cornea, as shown in Figure 6 and Figure 7. The entire measurement is completed in two seconds. In our study, recorded parameters of the anterior and posterior cornea were based on a best fit sphere (BFS) calculated for a fixed diameter of 8.0 mm and included corneal dioptric power parameters as well as astigmatism power and axis. The Pentacam system generates Zernike polynomial terms, a sequence of orthogonal polynomials that are used to quantify the extent of the individual aberrations and thus describe the shape of the aberrated wavefront. Zernike polynomials can either be displayed as surface-based elevation or as wavefront aberrations. The latter are available for the anterior, posterior and total cornea and are calculated by direct ray tracing [32]. Aberrations are divided into LOA (lower) and HOA (higher order aberrations). LOAs, defined as aberrations of the first and second order, include myopia, hyperopia and astigmatism and can easily be corrected by spectacles or refractive surgery. LOAs are responsible for most of the aberrations of the human cornea. HOAs, defined as aberrations of the third order and above, are more complex and only partially correctable. They account for up to 20% of corneal aberrations and are particularly disturbing to human vision when the pupil is dilated. For the analyzed HOAs, we limited ourselves to those of the 2nd to 4th order including secondary coma, as these are known to affect the visual quality the most [33]. Because the pterygium is a pathology of the anterior cornea in most cases, we focused mainly on the anterior and total corneal wavefront aberrations. Zernike coefficients and RMS (root mean square) values were calculated for a maximal pupil diameter of 6.0 mm. The following RMS values were analyzed: RMS of TA (total aberrations), RMS LOAs and RMS HOAs. Again, values from before and after irradiation were compared. In addition to analyzing the whole group, we analyzed a subgroup of pterygia with a horizontal length of 2 mm and more, since earlier findings showed a positive correlation between pterygium size and the number of aberrations [25,26,34]. In four cases, a comparison between several points in time was possible.

SPSS version 22 was used to perform statistical analysis. Due to the small number of cases, normal distribution could not be assumed. Thus, Wilcoxon signed-rank test and Friedman’s test (both non-parametrical tests) were used to compare data from before and after irradiation. Statistical significance was defined as *p* < 0.05.

## 3. Results

Orbscan data showed 9 pterygia had a mean horizontal length (HL) of 2.51 ± 0.78 mm (range 1.4–4 mm); no statistical significance was observed in the first 3 months after exclusive Strontium-/Yttrium-90 irradiation compared to pre-procedure measurements. Friedman’s analysis of variance of data from 5 pterygia with a HL > 2 mm (mean 3.0 ± 0.62 mm, range 2.4–4 mm) measured before irradiation and in the periods of the first 3 months and of 5 to 6 months after irradiation showed increasing with-the-rule astigmatism (mean +0.7 dpt ± 0.3, *p* = 0.016). Changes of corneal irregularity values in the central 3- and 5-mm zone were not statistically significant.

**Figure 8:**
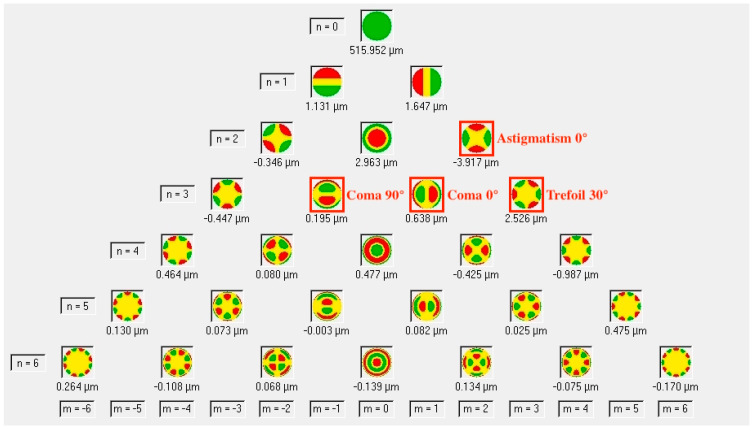
Zernike polynomials as displayed by the Pentacam software. The red framed aberrations increased significantly after exclusive Strontium-/Yttrium-90 beta irradiation of primary pterygia. The majority of HOAs (higher order aberrations) of the 3rd order, but still the overall RMS (root mean square) for HOAs of the 3rd to 6th order showed no statistical significance.

The main interest of this study was the interpretation of wavefront aberration data from Pentacam tomographies for its display of wavefront aberration data. Wilcoxon signed-rank test comparing tomographies of 11 pterygia with a mean HL of 2.02 mm ± 0.76 mm (range 1–3.5 mm) from before and within 1 year (mean 3.9 ± 4 months) after irradiation showed a significant increase in vertical coma for both total (+0.06 ± 0.21, *p* = 0.045) and anterior corneal wavefront aberrations (WFA) (+0.08 ± 0.01, *p* = 0.041), as seen in Figure 8. In addition, a significant reduction in horizontal pterygium length was confirmed (mean −0.26 ± 0.23 mm, *p* = 0.027).

Comparing only pterygia with a horizontal size of 2 mm or more (mean 2.52 ± 0.53 mm, range 2–3.5 mm, n = 6) showed an increase in RMS (root mean square) for both total corneal WFA (+0.59 ± 0.49, *p* = 0.027) and LOAs (+0.47 ± 0.41, *p* = 0.028). This was the same for anterior corneal WFA and total corneal WFA and may be explained by increasing anterior corneal horizontal astigmatism (+0.47 ± 0.21 dpt, *p* = 0.043).

Friedman’s analysis of variance of data from 4 pterygia with a horizontal length of 2 mm or more (mean 2.53 ± 0.61 mm, range 2–3.5 mm) measured before irradiation and in periods of the first 3 months and between 4 and 7 months after irradiation showed faint but continuous decrease in pterygium size (mean −0.6 ± 0.37 mm). Horizontal coma showed continuous increase for both total (+0.30 ± 0.09, *p* = 0.039) and anterior corneal WFA (+0.38 ± 0.01, *p* = 0.039). Interestingly, trefoil 30° of the anterior cornea increased (+0.26 ± 0.16, *p* = 0.018) in the first 3 months after irradiation but showed lower values again at later measurement points (overall +0.07 ± 0.06). RMS of LOAs of the anterior cornea increased as well (+0.32 ± 0.2, *p* = 0.39), while the other RMS values including RMS for higher order aberrations (HOAs 3rd to 6th order) showed no statistical significance.

In our study, no complications occurred in any patient after Ytt-/Sr-90 beta irradiation. Only a protracted redness in the pterygium area was observed in the first 3 months after treatment.

## 4. Discussion

Lateral UV light is deflected nasally by multiple reflections in the cornea at the two interfaces. This prismatic effect amplifies the energy of the 290–400 nm light by a factor of 20 compared to direct exposure [13]. It is suspected that this effect leads to nasal actinic damage of the limbal stem cells, which are still undifferentiated and show active mitotic cell division. The focal stem cell dysfunction can no longer guarantee the formation of corneal epithelial cells for the affected area. In this case, the conjunctival tissue, with Bowman’s membrane as its leading front, overcompensates and creates an aberrant structure.

**Figure 9:**
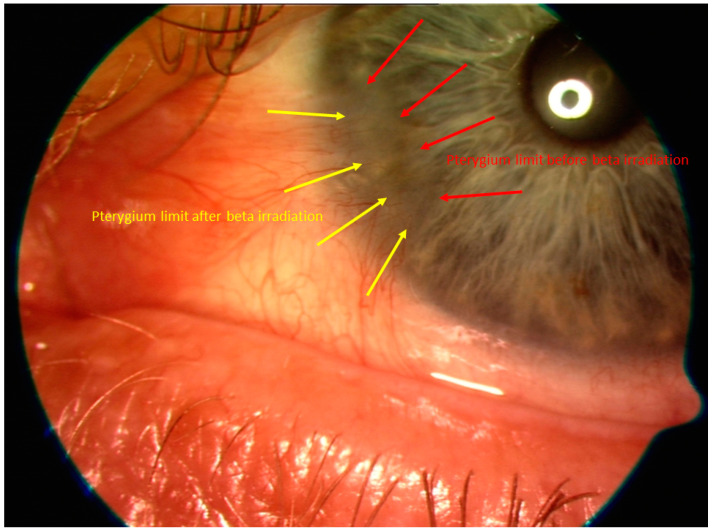
Shown is the regression of the pterygium after Sr-/Ytt-90 beta irradiation. The red arrows indicate where the border of the pterygium was before irradiation and the yellow arrows mark the border 12 months after irradiation.

Physiologically and radiologically, fibroblast cells are vulnerable to radiation-induced cell damage during their greatest mitotic activity [35]. This can be used therapeutically. After application of beta irradiation, vascularization and thus pterygium activity decreased continuously and significantly in our study until an avascular, flat, grey membrane remained, as seen in Figure 9.

Primary fibroblasts are inhibited by the radiobiological effect with secondary obliteration of the vessels of the pterygium. Depending on the size of the pterygium, this leads to induction of astigmatism and aberration.

Although excision and coverage with conjunctival autograft in combination with adjuvant application of antiproliferative substances or irradiation is currently considered the state-of-the-art therapy for pterygia, exclusive Strontium-/Yttrium-90 beta irradiation has shown to be an effective and, to date, a recurrence-free treatment principally requiring no surgery [18,23]. Pterygia are known to induce increasing astigmatism and HOA (higher order aberrations) when growing onto the cornea, thus affecting visual quality [25,26,36,37]. Different studies have shown a reduction in these aberrations after excision of the pathological tissue [38,39,40].

An important point to mention is what total irradiation dosage and fractions are required according to the latest aspects for beta irradiation of a pterygium. It has been shown by cellular and histological in vivo analysis that the target cell or tissue to be treated, which is important for the indication of targeted radiotherapy, is defined by the fibroblasts [35].

It could be shown that radiobiologically, a tissue tolerates a higher fractionated total dose than its application in a single dose, which is why fractionated irradiation is preferable to a high single dose with regard to late reactions. The total dose is important for the inactivation of the target cell. Therefore, the optimal total dose and fractions are calculated using isoeffect curves. The isoeffect curve can be calculated with the linear–quadratic formula (LQ). In vivo experiments have shown that when multiple fractions are applied, each has the same effect. This results in an optimal therapy ratio between prevention of recurrence and late complications by increasing the fractionation. Based on these data, the isoeffect curve is drawn up, where an optimal therapy tactic has been found with a total dose of 36Gy in 6 fractions for primary pterygia and 48 Gy in 8 fractions for recurrent pterygia [3]. There is a general consensus, also in recent studies, that a total dose of between 30 and 40 Gy in 5–8 fractions should be aimed for, which represents an optimal therapeutic ratio between treatment success and side effects [41,42,43].

Unfortunately, pterygia treated by exclusive Strontium-/Yttrium-90 beta irradiation can induce a further increase in astigmatism, which again correlates positively with their horizontal length (HL) prior to irradiation, probably due to pulling forces of the regressing pterygium [22,28]. The observations of this study support this theory, as both increasing horizontal astigmatism as well as decreasing horizontal length of pterygium size could be shown.

To our knowledge, there is no other research on the behavior of ocular aberrations other than astigmatism after exclusive Strontium-/Yttrium-90 beta irradiation in eyes with primary pterygia, so direct comparison is not possible. We focused on the wavefront aberrations of the anterior and total cornea because pterygia rarely affect the posterior cornea [44]. Applegate et al. showed that in HOAs, the central modes of the Zernike coefficients like coma and spherical aberration have the biggest impact on visual quality [33]. Whilst coma and trefoil aberrations have repeatedly found to be increased in eyes with pterygia, this seems not to be the case with spherical aberration [25,26,36]. As mentioned in the results, both vertical and horizontal coma were increased and likely influenced visual quality. We could not detect any changes in spherical aberration, but the subgroup analysis with different measuring points showed that horizontal coma increased continuously over time within the first 7 months after irradiation. Transient increase of trefoil 30° of the anterior cornea was only seen in pterygia with a HL > 2 mm in the first 3 months and might be explained by temporary inflammatory response due to irradiation. Despite these findings, RMS for total HOAs of the 3rd to 6th order did not show significant changes. On the other hand, RMS of LOAs increased what we believe to be explained by the increase in horizontal astigmatism.

We found that in pterygia of less than 2 mm horizontal length, HOAs do not increase significantly upon irradiation, and the imaging quality of the eye remains unchanged. For this reason, early Sr-/Ytt-90 beta irradiation is recommended. In pterygia of more than 2 mm horizontal length, an increase in certain HOAs was observed, i.e., the horizontal and vertical coma, as well as trefoil of the 3rd order of Zernike polynomials. For these reasons, we recommend that a secondary surgical excision of the pterygium be considered if the pterygium was greater than 2 mm before beta irradiation and if, at the same time, the HOAs increased significantly after Sr-/Ytt-90 application. We suggest performing the excision only after 6 months, as most of the regression of the pterygium takes place in the first 3 months after irradiation [22]. After irradiation, we found a different dynamic in the pterygium excision than in that of a non-irradiated primary pterygium. The inactive residual tissue of the pterygium can only be practically excised by peeling it out. A clear pterygium bed remains on the cornea. In rare cases, excision of the inactive avascular tissue from smaller pterygia was performed because of ongoing irritation or for cosmetic reasons. So far, no recurrence has occurred in either case. When pterygia have initial HL of <2 mm, exclusive Strontium-/Yttrium-90 beta irradiation thus remains an efficient and elegant way of treatment.

In addition to the recurrence rate, the complication rate is an important success criterion, which depends on the application technique, fractionation and dosage in postoperative irradiation. In our study, no complications occurred except for a slightly protracted conjunctival irritation for about 3 months. This postoperative process was observed in all patients.

The limitation of this study is its low case numbers. This is due to the fact that pterygia are not a common sight in our latitudes. The current assumption is that secondary excision reduces corneal aberrations due to other studies related to the conventional treatment method (conjunctival autograft + adjuvant therapy). It would therefore be of great interest to see whether the corneal aberrations in operated eyes after exclusive Strontium-/Yttrium-90 beta irradiation differ from those in eyes operated conventionally.

## 5. Conclusions

Exclusive Strontium/Yttrium-90 beta irradiation of the pterygium is a very effective and, to date, recurrence-free and safe treatment method against primary or recurrent pterygia.

For pterygium treatment management, it is advisable though to start irradiation as early as possible with a horizontal pterygium length of less than 2 mm before the induction of significant corneal aberrations by further pterygium growth make a second surgical excision necessary.

## Data Availability

The data presented in this study are available on request from the authors, in particular the datasets are archived in the clinics treated. The data are not publicly available as they contain information that could compromise the privacy of the participants.
